# Supplementary material for: A reproducible entropy-weighted Gower-CLARA-TOPSIS procedure for mixed-type MSME segmentation and prioritization
Source: MethodsX. 2026 Sep 18;17:104170. doi: 10.1016/j.mex.2026.104170 (PMC13632128; doi:10.1016/j.mex.2026.104170)
Supplement: Supplementary file 1 [file mmc2.pdf]

v b

MethodsX_Revised_R2 FIX Yeni UPLOAD NO TRACK

ARTIKEL 1

Document Details

Submission ID

trn:oid:::3618:152002615

Submission Date

Sep 17, 2026, 10:02 AM GMT+7

Download Date

Sep 17, 2026, 10:06 AM GMT+7

File Name

MethodsX_Revised_R2 FIX Yeni UPLOAD NO TRACK.docx

File Size

2.5 MB

16 Pages

6,764 Words

43,268 Characters

13% Overall Similarity

The combined total of all matches, including overlapping sources, for each database.

Filtered from the Report

- Bibliography

Match Groups

60 Not Cited or Quoted 11%
Matches with neither in-text citation nor quotation marks

6 Missing Quotations 1%
Matches that are still very similar to source material

3 Missing Citation 0%
Matches that have quotation marks, but no in-text citation

0 Cited and Quoted 0%
Matches with in-text citation present, but no quotation marks

Top Sources

11% Internet sources

7% Publications

0% Submitted works (Student Papers)

Integrity Flags

0 Integrity Flags for Review

Our system's algorithms look deeply at a document for any inconsistencies that would set it apart from a normal submission. If we notice something strange, we flag it for you to review.

A Flag is not necessarily an indicator of a problem. However, we'd recommend you focus your attention there for further review.

Match Groups

- **60** Not Cited or Quoted 11%
  Matches with neither in-text citation nor quotation marks
- **6** Missing Quotations 1%
  Matches that are still very similar to source material
- **3** Missing Citation 0%
  Matches that have quotation marks, but no in-text citation
- **0** Cited and Quoted 0%
  Matches with in-text citation present, but no quotation marks

Top Sources

11% Internet sources
7% Publications
0% Submitted works (Student Papers)

Top Sources

The sources with the highest number of matches within the submission. Overlapping sources will not be displayed.

#	Type	Source	%
1	Internet	github.com	4%
2	Internet	www.researchgate.net	<1%
3	Internet	mdpi-res.com	<1%
4	Publication	LaShell, Patrick Jonathon. "What's in a rule: Two-dimensional rule use in category...	<1%
5	Internet	metricgate.com	<1%
6	Internet	jamu-journal.ipb.ac.id	<1%
7	Internet	www.mdpi.com	<1%
8	Publication	Wumin Ouyang, Zhaohui Fan, Yong Han, Zihuan Wang, Yuqi Chen, Yuyu He, Hemi...	<1%
9	Publication	Gülşah Çelik Gül, Metin Gül. "From resource to innovation: A decision framework ...	<1%
10	Internet	pzs.dstu.dp.ua	<1%

11 Internet
d.docksci.com <1%

12 Internet
repository.uin-suska.ac.id <1%

13 Internet
www.freseniusmedicalcare.com <1%

14 Internet
iapress.org <1%

15 Internet
jeeemi.org <1%

16 Publication
A. Majumdar, B. Sarkar, P. K. Majumdar. "Determination of quality value of cotton... <1%

17 Internet
fs.unm.edu <1%

18 Internet
journal.globresco.com <1%

19 Publication
Ana-Maria Ifrim, Ionica Oncioiu. "A Hybrid Numerical–Semantic Clustering Algorit... <1%

20 Publication
Sathiyanesan Subanesam, Muthupandi Kalaiarasan, Neethirajan Neethiselvan, N... <1%

21 Internet
journal.trunojoyo.ac.id <1%

22 Internet
pmc.ncbi.nlm.nih.gov <1%

23 Internet
www.yumpu.com <1%

24 Internet
www2.mdpi.com <1%

25 Publication

Hasta Pratama, Fetty Fitriyanti Lubis, Jaka Sembiring. "AUFS-Samba: Adaptive Un... <1%

26 Internet

addi.ehu.es <1%

27 Internet

jurnal.ubhinus.ac.id <1%

28 Internet

karyailham.com.my <1%

29 Publication

Dušan Teodorović, Shinya Kikuchi, Djordje Hohlačov. "A routing and scheduling ... <1%

30 Internet

arxiv.org <1%

31 Internet

nawalaeducation.com <1%

32 Internet

www.fcn.eonerc.rwth-aachen.de <1%

33 Publication

Cindy Cahyaning Astuti, Bambang Widjanarko Otok, Shofi Andari. "Segmentation ... <1%

34 Publication

Ilias Politis, Lampros Dounis, Tasos Dagiuklas. "H.264/SVC vs. H.264/AVC video qu... <1%

35 Publication

Ahmad Daryanto. "An Introduction to Quantitative Research Methods for Market... <1%

36 Publication

Shengjie Wang, Shijun Lei, Mingjun Zhang, Catherine Hughes, Jagoda Crawford, Z... <1%

37 Publication

Xinzhi Du. "A Robust and High-Dimensional Clustering Algorithm Based on Featur... <1%

MethodsX
Open access
Article Template

Article information

Article title

A Reproducible Entropy-Weighted Gower-CLARA-TOPSIS Procedure for Mixed-Type MSME Segmentation and Prioritization

Authors

Yeni Kustiyahningsih[1], Eza Rahmanita[1], Miswanto[2], Imamah[1], Herdiyanti Fifin Purwaningrum[1], Firdausi Putri Cahyani[1], Deshinta Arrova Dewi[3], Bain Khusnul Khotimah[4], Jaka Purnama[5]

Affiliations

[1]Department of Information Systems, Faculty of Engineering, Universitas Trunodjoyo Madura, Bangkalan 69162, East Java, Indonesia

[2]Department of Mathematics, Faculty of Science and Technology, Universitas Airlangga, Surabaya 60115, East Java, Indonesia

[3]Department of Computer Science and Technology, Faculty of Information Technology, INTI International University, Malaysia

[4]Department of Informatics, Faculty of Engineering, University of Trunodjoyo Madura, Bangkalan, East Java 69162, Indonesia

[5]Department of Industrial Engineering, Faculty of Engineering, Universitas 17 Agustus 1945 Surabaya 60118, East Java, Indonesia

Corresponding author’s email address and Twitter handle

ykustiyahningsih@trunojoyo.ac.id

Keywords

Mixed-type data; Gower distance; CLARA; entropy weighting; feature selection; TOPSIS; MSME segmentation

Related research article

Firmansyah, M. I., Kustiyahningsih, Y., Rahmanita, E., Abidin, M. S., & Satoto, B. D. (2025). Optimization of MSMEs clustering in Sampang District using K-Medoids and the Silhouette Coefficient. Teknika, 14(1), 1–8. https://doi.org/10.34148/teknika.v14i1.1116. The related article reports an application study; the present MethodsX article provides a distinct, reusable procedure that extends the workflow through entropy-weighted Gower dissimilarity, CLARA, Top-N feature selection, and within-cluster TOPSIS prioritization.

For a published article

Not applicable.

Abstract

In this paper a reproducible approach for segmentation of mixed-type data and the within-cluster prioritizing is presented. The procedure keeps raw categorical values for Gower dissimilarity, binary or count representations only for entropy weighting, applies CLARA to repeated samples with PAM medoids, and evaluates weighted and Top-N feature configurations with the Silhouette Coefficient, medoid-based Davies-Bouldin Index, and Calinski-Harabasz Index. The chosen configuration is then utilized to rank within clusters, instead of between clusters using TOPSIS to evaluate the economic-operational preparedness of similar firms. It presents a worked example with 1,049 MSME records from Sampang, Indonesia, with numerical, binary, count and categorical attributes. The optimum configuration selected four operational and financial aspects and increased the Silhouette Coefficient from 0.4183 to 0.5588, decreased Davies-Bouldin Index from 1.2393 to 0.6181, and boosted the Calinski-Harabasz Index from 357.7787 to 992.3958. By combining entropy-based feature weighting, weighted Gower dissimilarity,

ELSEVIER
MethodsX
Open access
Article Template

CLARA sampling, and Top-N feature selection, the procedure provides transparent mixed-data segmentation while preserving categorical semantics and separating clustering from peer-group-based TOPSIS ranking. The procedure supports SDG 8, SDG 9, and SDG 12. A reference Jupyter Notebook implementation and an anonymized or de-identified demonstration dataset are supplied to support inspection and reuse of the workflow.

Graphical abstract

The graphical abstract presents the proposed Entropy-Weighted Gower-CLARA-TOPSIS procedure as a reproducible workflow for mixed-type MSME segmentation and within-cluster prioritization. The workflow begins with cleaned MSME data containing numerical, binary, count, and categorical variables. Categorical values are preserved for Gower dissimilarity, while suitable binary, count, or numerical representations are used to calculate entropy-based feature weights. The weighted Gower distance matrix is then used in CLARA clustering, followed by validation using the Silhouette Coefficient, medoid-based Davies-Bouldin Index, and Calinski-Harabasz Index. After the best cluster configuration is selected, TOPSIS is applied separately within each cluster to produce an economic-operational readiness ranking among comparable MSMEs.

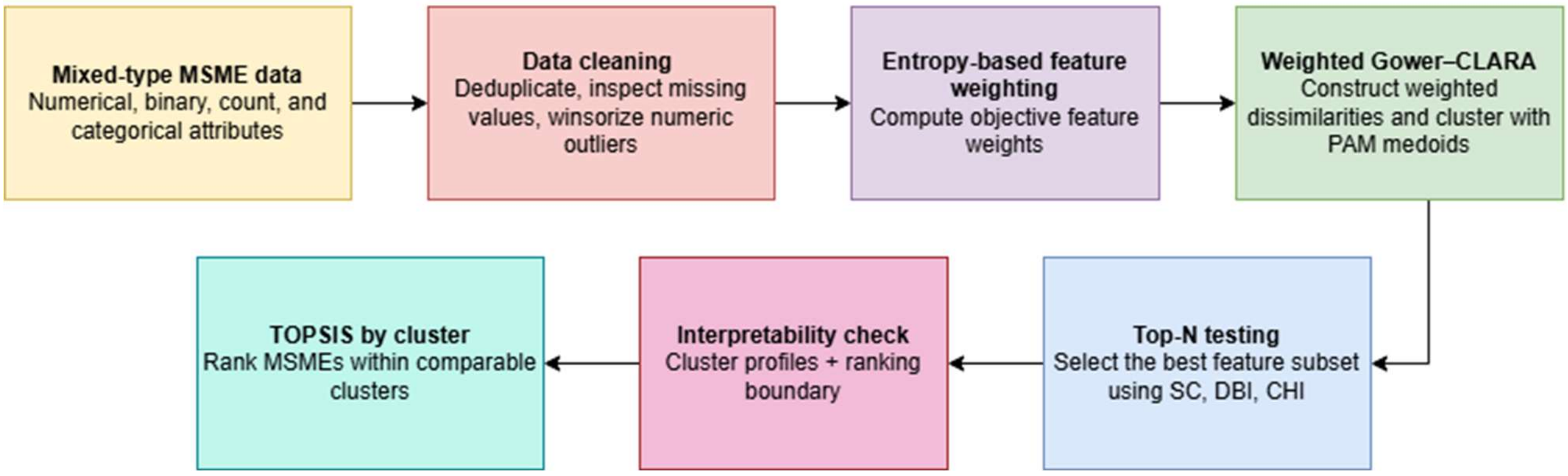

Figure 1. Graphical abstract of the proposed Entropy-Weighted Gower-CLARA-TOPSIS procedure for mixed-type MSME segmentation and within-cluster prioritization.

Specifications table

Subject area	*Computer Science*
More specific subject area	*Mixed-type clustering, decision support systems, and multi-criteria decision making.*
Name of the method	*Entropy-Weighted Gower-CLARA-TOPSIS (EWGCT) procedure.*
Name and reference of original method	*PAM and CLARA medoid clustering [1], [2]; entropy weighting [3], [4], [5]; TOPSIS [6].*
Resource availability	*The complete reproducibility package is available at: https://github.com/yenikustiyahningsih/EWGCT-MSME-Segmentation*
Primary data type	*Cross-sectional mixed-type organizational records: continuous, count, binary, and nominal variables.*
Method output	*Entropy weights; selected features; precomputed weighted Gower matrix; CLARA medoids and labels; internal validation metrics; within-cluster TOPSIS ranks.*
Intended use	*Mixed-type MSME segmentation, within-cluster prioritization, regional enterprise profiling, and data-driven decision support for MSME development.*

ELSEVIER
MethodsX
Open access
Article Template

Related SDGs	*SDG 8: Decent Work and Economic Growth; SDG 9: Industry, Innovation and Infrastructure; SDG 12: Responsible Consumption and Production.*

Background

Decision-support datasets in enterprise and regional development commonly contain heterogeneous variables, including continuous financial indicators, count-based operational measures, binary legal indicators, and nominal business attributes. MSME data are a clear example because firm size, assets, revenue, workforce, production capacity, formal registration, digital-platform use, and ownership characteristics are often recorded in different measurement forms. To address this heterogeneity, the procedure draws on three complementary methodological components: partitioning-around-medoids and CLARA-based medoid clustering for mixed-type data [1], [2], objective entropy-based weighting to reflect differing attribute informativeness [3], [4], [5], and TOPSIS-based closeness ranking of alternatives [6]. Earlier SME studies have shown that small and medium enterprises are important for employment and economic activity across countries [7] ,while clustering-based MSME studies show that data-driven segmentation can support more targeted profiling and development planning [8], [9].

Mixed-type data require special treatment of distances. Mostly traditional Euclidean clustering is suited to numeric variables and can give misleading distances if category variables are coerced into numeric codes. Gower's coefficient is a typical solution since it integrates feature-level dissimilarities for numerical, binary and categorical data into one comparable distance framework [10]. Recent studies of mixed-data clustering further point out that real-world datasets usually contain both continuous and categorical features, therefore the design of mixed-type similarity or distance is fundamental to accurate segmentation [11], [12], [13]. PAM based medoid clustering may be computationally expensive for bigger datasets. Therefore, CLARA expands k-medoids by searching the medoids from repeated samples and evaluating prospective medoids on the whole dataset [1], [2].

However, mixed-data segmentation often treats all variables identically, regardless of their variability and discriminative power. This can impair the interpretability of the clusters, especially if variables with low information dominate or dilute the distance structure. The entropy weighting method is an objective method for obtaining feature weights based on information dispersion and it is based on Shannon entropy theory [3]. Recent studies on entropy based similarity assessment for mixed numerical and categorical data also support the use of entropy weighting to indicate varied attribute relevance in clustering [4]. The method preserves raw categorical semantics while computing Gower dissimilarity and applies entropy weighting exclusively to relevant numeric, count or binary representations.

Following segmentation, decision makers generally want not only cluster labels but also a visible approach to prioritize entities within comparable peer groups [14]. TOPSIS is suitable for this stage as it rates the options based on their relative closeness to positive and negative ideal solutions. This approach is therefore a combination of entropy weighing, weighted Gower dissimilarity, CLARA clustering, Top-N feature evaluation, and within-cluster TOPSIS ranking. The resulting procedure is designed to offer reproducible MSME segmentation and economic-operational readiness prioritization without direct comparison of the TOPSIS scores across structurally diverse clusters [14].

Method details

A. Method overview and prerequisites

The EWGCT method provided here is a reproducible approach for mixed-type MSME segmentation and within-cluster prioritizing. The process starts with mixed-type data of MSMEs having numerical, count, binary and nominal variables. Prior to clustering, the data are cleaned and documented by removing duplicates, inspecting for missing values, addressing outliers, and documenting preprocessing decisions. Then the procedure continues with a dual variable representation approach, where raw category data are retained for Gower dissimilarity, but meaningful numerical, binary or count representations are created for entropy weighting. The feature weights based on entropy are then incorporated in the weighted Gower dissimilarity matrix that is employed for CLARA clustering and model evaluation.

MethodsX
Open access
Article Template

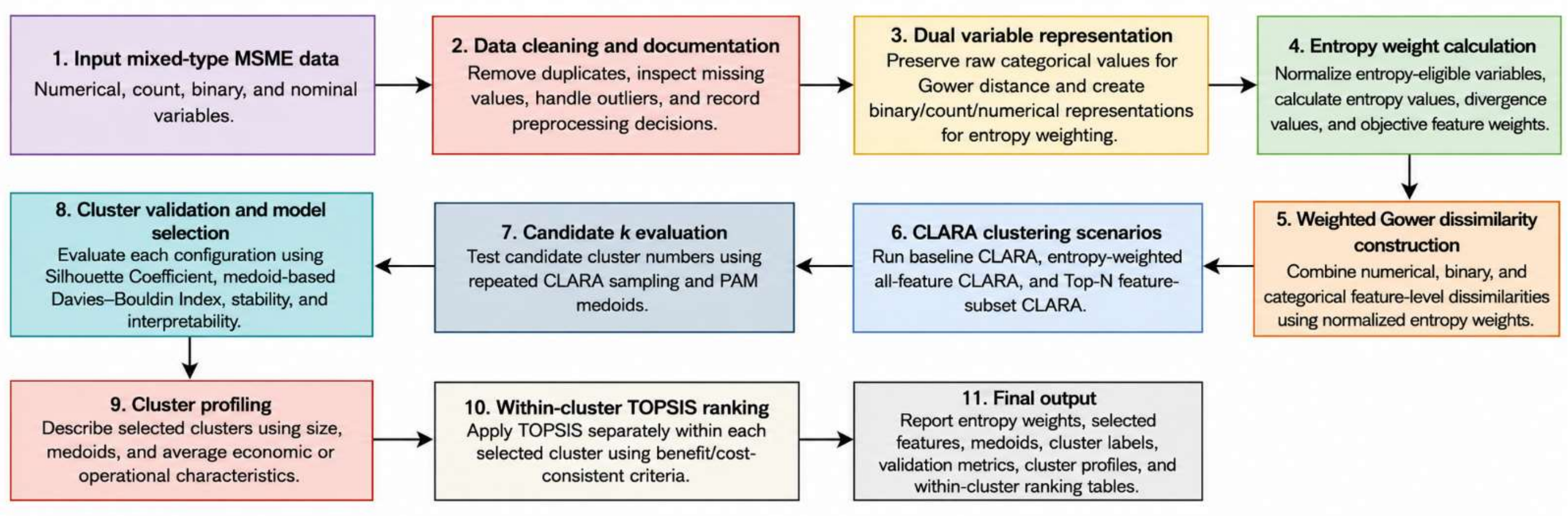

Figure 2. Workflow of the Entropy-Weighted Gower–CLARA–TOPSIS method for MSME segmentation and ranking.

As illustrated in Figure 2, the workflow comprises eleven operational steps. After the construction of the weighted Gower dissimilarity matrix, the process assesses a number of clustering scenarios, including baseline CLARA, entropy-weighted all-feature CLARA, and Top-N feature-subset CLARA. Candidate numbers of clusters are examined with repetitions of CLARA sampling and PAM medoids and are evaluated by Silhouette Coefficient, medoid-based Davies– Bouldin Index, stability, and interpretability. The cluster profiles are examined after the final clustering solution has been chosen to facilitate substantive interpretation. Finally, comparable MSMEs are analyzed separately in each of the identified clusters using TOPSIS. The final outputs are entropy weights, selected features, medoids, cluster labels, validation metrics, cluster profiles, and within-cluster ranking tables.

B. Data specification and dual variable representation

Let X contain n entities and p clustering features. Partition the features into numerical or count features N and categorical features C. The raw representation is retained for distance calculation. For entropy weighting, the same categorical features are represented only through a defensible binary or count encoding. In the worked example, legal registration and land ownership are binary, whereas social-media and marketplace adoption are platform counts. This avoids treating arbitrary category codes as quantitative values while still allowing entropy to quantify the observed dispersion of a pre-defined, meaningful indicator.

Table 1. *Rules for the dual representation of mixed-type variables.*

Feature type	***Use in weighted Gower distance***	***Use in entropy matrix***	***Example in worked demonstration***
Continuous / monetary	*Range-normalized absolute difference*	*Min-Max normalized value*	*Revenue, assets*
Count	*Range-normalized absolute difference*	*Min-Max normalized count*	*Workforce, production capacity, number of platforms*
Binary	*0 if equal; 1 if different*	*0/1 indicator*	*Formal business registration availability, Land ownership*
Nominal	*0 if equal; 1 if different*	*Not directly coded unless a meaningful binary/count transformation exists*	*Nominal categories retained as raw values in Gower; not used for entropy unless a defensible binary/count transformation exists*

C. Data preparation

ELSEVIER
MethodsX
Open access
Article Template

Before calculating any dissimilarity matrix, do the following steps:

1. Remove duplicate entity records via a documented identifier or a documented duplicate-matching rule, to avoid repeated observations affecting cluster generation [15].
2. Assess the missingness for all possible variables. In the presence of missing values, utilize a justified imputation method or a Gower implementation that takes into account feature-level missingness. For the mixed-type data, non-parametric imputation can be considered when both numerical and categorical variables are present [16].
3. Inspect numeric outliers using the interquartile range (IQR) rule. To limit the leverage of extreme values while retaining observations, winsorize values that fall outside the lower and upper IQR fences [17]. The IQR is calculated as:

$$IQR = Q3 - Q1 \quad (1)$$

where $(Q1)$ and $(Q3)$ denote the first quartile (25th percentile) and the third quartile (75th percentile), respectively. The lower and upper IQR fences are then defined as:

$$Lower\ fence = Q1 - 1.5 \times IQR$$
$$Upper\ fence = Q3 + 1.5 \times IQR \quad (2)$$

Values below the lower fence or above the upper fence are treated as potential outliers and are winsorized to the corresponding fence values.

4. For numeric variables, use Min-Max scaling only in the entropy matrix to create non-negative and comparable values. Gower distances independently range-normalize each numeric feature, so this scaling is not used as a replacement for mixed-type distance handling [10], [18]. For an observation (i) and feature (j), the Min-Max normalized value is calculated as:

$$x_{ij}' = \frac{x_{ij} - x_{j,min}}{x_{j,max} - x_{j,min}} \quad (3)$$

where (x_{ij}) is the original value of observation (i) for feature (j), (x_{ij}') is the corresponding Min-Max normalized value, $(x_{j,min})$ and $(x_{j,max})$ are the minimum and maximum observed values of feature (j), respectively, $(i = 1, \dots, n)$ denotes the observation index, and $(j = 1, \dots, p)$ denotes the feature index.
Equation (3) is used only to create a non-negative and comparable entropy matrix. It is not a replacement for mixed-type distance handling.

5. Record every threshold, transformation, random seed, and exclusion decision in a reproducibility log to support transparent and repeatable computational analysis [19].

D. Objective entropy weighting

For each entropy-eligible feature (j), create a non-negative normalized value x_{ij}. To avoid $log(0)$, add a small constant ε, for example 10–12, only in the probability calculation. Entropy weighting is used to derive objective feature weights from the information dispersion of each variable, following Shannon's entropy principle and its use in multi-attribute decision-making [3], [5]. This weighting strategy is also suitable for mixed-data clustering because it can represent different levels of attribute importance before distance calculation [4]. The normalized proportion for observation (i) and feature (j) is calculated as:

$$p_{ij} = \frac{x_{ij} + \varepsilon}{\sum_{k=1}^{n} x_{kj} + \varepsilon} \quad (4)$$

where (x_{ij}) denotes the non-negative normalized value of observation (i) for feature (j), (ε) is a small positive constant used only to avoid $(\ln 0)$, (n) is the number of observations, and (k) is a dummy summation index. The index $(i = 1 \dots, n)$ denotes the observation index, while (j) denotes the feature index.

The entropy value of each feature is then calculated as:

$$E_j = -\frac{1}{\ln n} \sum_{i=1}^{n} p_{ij}\, ln(p_{ij}) \quad (5)$$

where (E_j) denotes the entropy value of feature (j).

The degree of diversification, or information utility, is obtained as:

$$d_j = 1 - E_j \quad (6)$$

where (d_j) denotes the information utility or degree of diversification of feature (j).

Finally, the objective entropy weight for each feature is calculated as:

MethodsX
Open access
Article Template
ELSEVIER

$$w_j = \frac{d_j}{\sum_{k=1}^{m} d_k} \quad (7)$$

where (w_j) denotes the resulting objective entropy weight of feature (j), (m) denotes the number of entropy-eligible features, and (k) is a dummy summation index over the entropy-eligible features.

A larger (w_j) indicates a feature with greater observed information dispersion under the declared representation. However, entropy weighting is objective only in the sense that it is derived from data variation; it does not establish substantive importance, policy relevance, or causal influence. Therefore, analysts should retain a pre-analysis rationale for candidate variables and inspect whether the resulting weights are plausible [20].

E. Weighted Gower dissimilarity

For a pair of entities (a) and (b), calculate a partial dissimilarity (δ) for each feature (j). For a numerical or count feature, the partial dissimilarity is calculated as the range-normalized absolute difference. For a categorical feature, the partial dissimilarity is 0 when the categories are equal and 1 otherwise. This follows the Gower dissimilarity framework for mixed-type data, which allows numerical, binary, and categorical variables to be combined in a single distance matrix [10], [21]. To reflect the different information contributions of variables, the entropy weights are incorporated into the Gower aggregation step [13]. The weighted Gower dissimilarity between entities (a) and (b) is calculated as:

$$D_{ab} = \frac{\sum_{j=1}^{p} w_j m_{abj} \delta_{abj}}{\sum_{j=1}^{p} w_j m_{abj}} \quad (8)$$

where (D_{ab}) denotes the weighted Gower dissimilarity between entities (a) and (b), (w_j) denotes the normalized entropy weight of feature (j), (m_{abj}) is a validity indicator that equals 1 when the pairwise comparison is valid and 0 when the corresponding feature value is missing, and (δ_{abj}) denotes the feature-specific partial dissimilarity between entities (a) and (b) for feature (j). The indices (a) and (b) denote the two entities being compared, (j) denotes the feature index, and (p) denotes the number of features included in the weighted Gower distance calculation.

In the complete demonstration dataset, ($m_{abj} = 1$) for all feature comparisons. The same feature order and normalized weights must be used consistently in every distance calculation for a given scenario.

F. CLARA with precomputed weighted distances

CLARA is a sampling-based extension of PAM that evaluates medoid candidates on the full dataset after each subset search [1], [2]. For each candidate (k), the complete weighted Gower dissimilarity matrix is calculated once per feature scenario. The procedure is repeated (b) times: (s) cases are randomly sampled without replacement, PAM is applied to the corresponding submatrix, the resulting medoids are mapped to their original row positions, and all (n) cases are assigned to the closest medoid using the full dissimilarity matrix. The medoid set with the lowest total full-data dissimilarity is retained. The sample size is determined as:

$$s = min\{n,\ max(50 + 5k,\ \lceil 0.25n \rceil)\} \quad (9)$$

where (s) denotes the number of cases sampled for each CLARA iteration, (n) denotes the total number of cases in the dataset, (k) denotes the candidate number of clusters. Here, $\cdot$ denotes the ceiling function, which rounds the enclosed value up to the nearest integer. Equation (9) defines the sample-size policy used in the worked demonstration. The policy was selected as a practical implementation choice to balance computational efficiency and sample coverage by increasing the sample size with the candidate number of clusters while ensuring that the sample includes at least 25% of the dataset when this yields a larger sample, subject to the full dataset size n. It is not intended to represent a universal CLARA rule. The demonstration uses $b = 5$ sampling iterations for each candidate $k = 2, \ldots, 10$, with random seed 42. When n is small relative to the sampling target, the minimum operator in Equation (9) caps s at n, so the CLARA subsample equals the full dataset and the procedure reduces to a single exhaustive PAM run on all n cases; because Equation (9) already bounds s by n, this edge case is handled automatically and requires no separate implementation logic. Future applications may evaluate alternative sample fractions, numbers of sampling iterations, or multiple random seeds when a robustness assessment is required.

G. Top-N feature selection and scenario design

Sort all candidate features by entropy weight in descending order. This unsupervised feature selection technique does not require class labels and attempts to increase the clustering quality and interpretability [22], [23]. Evaluate the complete weighted model (Top-(p)) and a descending sequence of Top-(N) subsets. Re-normalize the retained feature weights to total to 1 for each Top-(N) configuration. Feature reduction can eliminate less informative variables but retain useful clustering structure [24], [25]. The selected arrangement should give a high Silhouette Coefficient and low Davies-Bouldin Index while at the same time ensure stability and interpretability of clusters [26], [27]. A small improvement in an internal validation index alone should not suffice to justify the choice of an unstable or substantively uninterpretable configuration. The ordered feature set is given by :

$$S_m = \{f_{(1)}, f_{(2)}, \ldots, f_{(m)}\}, where\ w(f_{(1)}) \geq w(f_{(2)}) \geq \cdots \geq w(f_{(m)}) \quad (10)$$

where (S_m) denotes the ordered set of (m) candidate features, ($f_{(r)}$) denotes the feature ranked (r)-th by entropy weight, and $w(f_{(r)})$) denotes its corresponding entropy weight. The Top-(N) subset consists of the first (N) features in this ordered set.

H. Cluster validation

Use the Silhouette Coefficient (SC) to quantify within-cluster cohesion relative to the nearest alternative cluster [26]. For a precomputed dissimilarity matrix, calculate (a_i) as the mean dissimilarity of observation (i) to members of its own cluster and (b_i) as the minimum mean dissimilarity to members of another cluster:

$$SC(i) = \frac{b_i - a_i}{\max(a_i, b_i)} \quad (11)$$

where $SC(i)$ denotes the Silhouette Coefficient of observation i, a_i is the mean dissimilarity of observation i to members of its own cluster, and b_i is the minimum mean dissimilarity of observation i to members of another cluster.

Use a medoid-based Davies-Bouldin Index (DBI) for arbitrary dissimilarities [8]. Let (S_i) be the mean dissimilarity from members of cluster (i) to its medoid (m_i), and (M_{ij}) the dissimilarity between medoids (m_i) and (m_j). Then:

$$DBI = \frac{1}{K}\sum_{i=1}^{K} \underset{j \neq i}{max}\left(\frac{S_i + S_j}{M_{ij}}\right) \quad (12)$$

where DBI denotes the Davies-Bouldin Index, K is the number of clusters, S_i is the mean dissimilarity of members of cluster i to its medoid m_i, and M_{ij} is the dissimilarity between medoids m_i and m_j.

Use the Calinski-Harabasz Index (CH) to measure the ratio between between-cluster and within-cluster dispersion [9]. Let (B_k) and (W_k) denote the between- and within-cluster dispersion matrices [28], respectively:

$$CH = \frac{tr(B_k)}{tr(W_k)} \times \frac{n-1}{k-1} \quad (13)$$

where CH denotes the Calinski-Harabasz Index, B_k and W_k are the between- and within-cluster dispersion matrices, respectively, n is the number of observations, k is the number of clusters, and $tr(\cdot)$ denotes the matrix trace, i.e., the sum of the diagonal elements [28].

For each configuration, report (k), sample size, number of CLARA sampling iterations, random seed(s), selected features, entropy weights, total dissimilarity cost, SC, DBI, and CH. Interpret the selected configuration jointly based on cohesion, separation, compactness, stability, and cluster interpretability. When benchmark labels are available, an external validation metric may be added; otherwise, cluster stability across resamples or random seeds should be reported.

I. Within-cluster TOPSIS ranking

Apply TOPSIS only after selecting the final cluster model. TOPSIS ranks alternatives according to their relative closeness to positive and negative ideal solutions. In the demonstration, TOPSIS is applied separately within each cluster using four selected numeric criteria: production capacity, revenue, assets, and workforce. All four are treated as benefit criteria because the ranking represents economic-operational readiness. The criterion direction must be defined before determining the ideal solutions, as TOPSIS results depend on criterion direction and assigned weights. A needs-based intervention ranking requires a different criterion definition and cannot be inferred from this economic-operational readiness ranking. For each cluster, vector normalization is performed as:

$$r_{ij} = \frac{x_{ij}}{\sqrt{\sum_{k=1}^{n} x_{kj}^2}} \quad (14)$$

where (x_{ij}) is the value of alternative (i) for criterion (j), (r_{ij}) is its normalized value, and (n) is the number of alternatives within the cluster.

The weighted normalized matrix is then calculated as:

$$v_{ij} = w_j r_{ij} \quad (15)$$

where (v_{ij}) is the weighted normalized value and (w_j) is the normalized weight of criterion (j).

Because all criteria in the demonstration are benefit criteria, the positive and negative ideal solutions are defined as:

$$A^+ = \begin{cases} \max_i(v_{ij}), j \in J_b \\ \min_i(v_{ij}), j \in J_c \end{cases}$$
$$A^- = \begin{cases} \min_i(v_{ij}), j \in J_b \\ \max_i(v_{ij}), j \in J_c \end{cases} \quad (16)$$

where A^+ and A^- denote the positive and negative ideal solutions, respectively, J_b denotes the set of benefit criteria, and J_c denotes the set of cost criteria. In the present demonstration, all four criteria are benefit criteria; therefore, the positive ideal takes the maximum value and the negative ideal takes the minimum value for each criterion.

The separation of alternative (i) from the positive and negative ideal solutions is calculated as:

$$S_i^+ = \sqrt{\sum_{j=1}^{m} \left(v_{ij} - A_j^+\right)^2} \quad (17)$$
$$S_i^- = \sqrt{\sum_{j=1}^{m} \left(v_{ij} - A_j^-\right)^2}$$

where (S_i^+) and (S_i^-) are the distances of alternative (i) from the positive and negative ideal solutions, respectively, and (m) is the number of criteria.

The relative closeness coefficient is:

$$C_i = \frac{S_i^-}{S_i^+ + S_i^-} \quad (18)$$

where (C_i) denotes the relative closeness of alternative (i) to the positive ideal solution. A higher (C_i) indicates greater relative closeness to the positive ideal. Because normalization and ideal points are computed separately within each cluster, (C_i) scores should not be compared across clusters. Report each rank and score together with its cluster label and interpret the result as a within-cluster economic-operational readiness ranking.

J. Reference algorithm

```
INPUT: mixed-type data X; feature types; candidate k values; b CLARA samples; Top-N set; random seeds
1  Clean data; preserve raw categories for Gower; create entropy-eligible binary/count representations.
2  Compute entropy weights for all candidate features.
3  FOR each scenario in {baseline, weighted all-features, Top-N subsets}:
4      Construct scenario-specific weighted Gower matrix D.
5      FOR each k:
6          Repeat b times: sample s records; run PAM; score candidate medoids on full D.
7          Retain medoids with minimum full-data cost; calculate SC and medoid-based DBI.
8      END FOR
9  END FOR
10 Select the configuration using SC, DBI, CH, stability, and substantive interpretability.
11 Within each final cluster, execute benefit/cost-consistent TOPSIS and report only within-cluster ranks.
OUTPUT: weights, selected features, medoids, labels, validation metrics, within-cluster ranking tables.
```

K. Software implementation and reproducibility package

The accompanying Jupyter Notebook-based Python implementation uses pandas, NumPy, scikit-learn, scikit-learn-extra, scipy, matplotlib, seaborn, and openpyxl for data processing, PAM/CLARA clustering, clustering evaluation, visualization, spreadsheet handling, and reproducible computation [29], [30], [31]. The notebook implements data

turnitin Page 14 of 21 - Integrity Submission Submission ID trn:oid:::3618:152002615

preprocessing, entropy-based feature weighting, weighted Gower dissimilarity calculation, CLARA clustering with PAM medoid search, internal validation metrics, Top-N feature evaluation, and within-cluster TOPSIS ranking. The implementation is written explicitly to make each computational step inspectable rather than optimized for very large datasets. For substantially larger datasets, researchers should use memory-aware distance computation, blockwise processing, or a proven medoid implementation while preserving the stated distance, sampling, validation, and ranking specifications. The complete reproducibility package is available at: https://github.com/yenikustiyaningsih/EWGCT-MSME-Segmentation.

Table 2. *Contents of the reproducibility package.*

Supplementary file	***Purpose***	***Included material***
entropy_clara_topsis.ipynb	*Main executable notebook for the proposed EWGCT workflow.*	*Data preprocessing, entropy weighting, weighted Gower dissimilarity, CLARA clustering, validation metrics, Top-N feature selection, and within-cluster TOPSIS ranking.*
dataset_umkm_sampang_25new.xlsx	*Demonstration input dataset.*	*Anonymized or de-identified MSME sample data used to demonstrate the workflow.*
hasil_all_clustering_topsis.xlsx	*Combined clustering and TOPSIS output file.*	*Final clustering results and within-cluster TOPSIS ranking results.*
requirements.txt	*Python dependency list.*	*Required Python packages and versions for reproducing the computational workflow. Every dependency is pinned to an exact version using the package==version syntax (for example, pandas==2.3.3, numpy==1.26.4, scikit-learn==1.4.2, scikit-learn-extra==0.3.0), so the computational environment can be reproduced exactly rather than approximately.*
README_TOPSIS.md	*Basic usage instructions.*	*Instructions for running the notebook and interpreting TOPSIS outputs.*
TOPSIS_IMPLEMENTATION_GUIDE.md	*Technical implementation guide.*	*Detailed explanation of the TOPSIS procedure, criteria, weights, and ranking process.*

Supplementary materials supplied with this manuscript include the main Jupyter Notebook implementation, dependency specifications, reproducibility instructions, TOPSIS implementation guide, an anonymized/de-identified MSME dataset, preprocessing output, clustering output, and combined clustering–TOPSIS output. These files support inspection and reproduction of the proposed workflow. The supplementary package does not include direct business identifiers or the non-anonymized source records. All shared data have been anonymized/de-identified before submission.

Method validation

A. Worked demonstration: Sampang MSME dataset

turnitin Page 14 of 21 - Integrity Submission Submission ID trn:oid:::3618:152002615

The method was demonstrated using an administrative dataset of MSMEs in Sampang, Indonesia. The raw dataset contained 1,056 records. Seven duplicated records were removed, yielding 1,049 observations. The modelling inputs Formal business registration availability, workforce, annual production capacity, annual revenue, assets, social-media use, marketplace use, and land ownership. No missing value remained after cleaning. The original administrative dataset contained business-identifying fields. Before sharing, direct identifiers were removed or replaced, including owner names, business names, addresses, phone numbers, and other fields that could directly identify specific MSMEs. Therefore, the supplementary package contains an anonymized/de-identified version of the empirical dataset used in the worked demonstration, not the non-anonymized source records.

Table 3. *Input variables used in the worked demonstration.*

Variable	Raw type	Distance treatment	Entropy representation
Formal business registration availability	Binary categorical	Match/mismatch	0 = unavailable; 1 = available
Workforce	Count	Range-normalized absolute difference	Min-Max normalized count
Annual production capacity	Count/continuous	Range-normalized absolute difference	Min-Max normalized value
Annual revenue	Continuous monetary	Range-normalized absolute difference	Min-Max normalized value
Assets	Continuous monetary	Range-normalized absolute difference	Min-Max normalized value
Social-media use	Count	Range-normalized absolute difference	Number of platforms
Marketplace use	Count	Range-normalized absolute difference	Number of platforms
Land ownership	Binary	0 if equal; 1 if different	0 = non-owned; 1 = owned

B. Entropy weights

The entropy procedure placed the highest weights on production capacity (0.2424), assets (0.1744), annual revenue (0.1719), and workforce (0.1672). The lowest weight was assigned to Formal business registration availability (0.0169). Figure 3 presents the feature weights. The result is interpreted as observed discriminative information in this data configuration, not as an assessment of policy importance.

ELSEVIER
MethodsX
Open access
Article Template

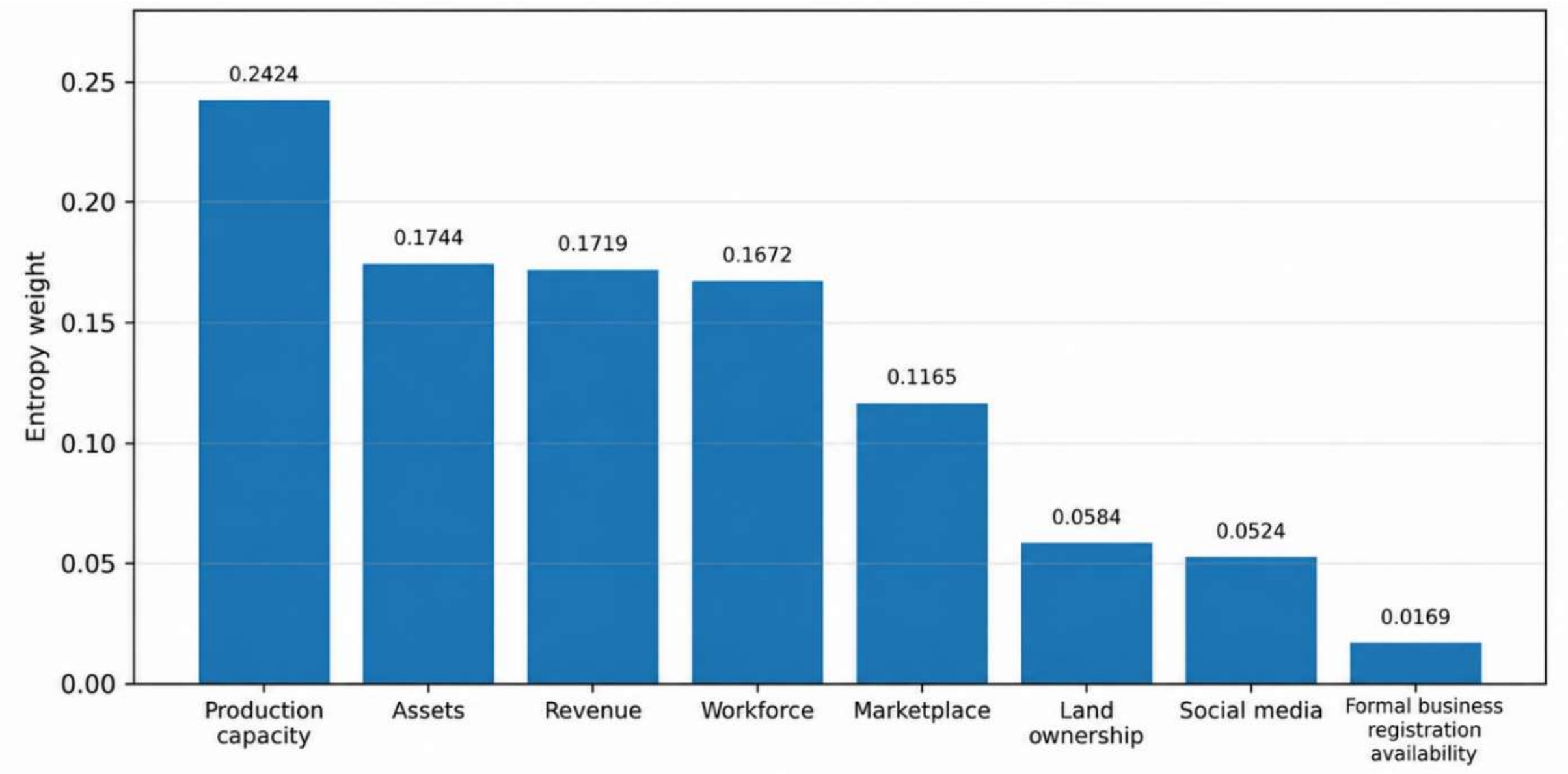

Figure 3. *Entropy weights obtained in the worked demonstration.*

C. Baseline and weighted CLARA comparison

Table 4 compares the baseline (unweighted) CLARA model with the entropy-weighted CLARA model across k = 2–10 using the Silhouette Coefficient, medoid-based Davies-Bouldin Index, and Calinski-Harabasz Index. For the baseline model, the highest Silhouette Coefficient, the lowest DBI, and the highest CH value all occurred at k = 4. This indicates that k = 4 provided the most consistent baseline solution in terms of cohesion, separation, and compactness. For the weighted model, the highest Silhouette Coefficient occurred at k = 4, the lowest DBI occurred at k = 2, and the highest CH value occurred at k = 3. Under the pre-specified selection rule, k = 4 was retained because it maximized the Silhouette Coefficient, while DBI and CH were used as complementary validation indicators. At k = 4, entropy weighting improved the Silhouette Coefficient from 0.4183 to 0.4637 and reduced the medoid-based DBI from 1.2393 to 0.9258. Although the CH value decreased from 357.7787 to 253.9949 at k = 4, the weighted model was still retained because the main objective was to improve distance-based cluster cohesion and separation under the weighted Gower–CLARA framework.

Table 4. *Baseline and entropy-weighted CLARA validation results.*

k	*Baseline SC*	*Baseline DBI*	*Baseline CH*	*Weighted SC*	*Weighted DBI*	*Weighted CH*
2	*0.3086*	*2.0058*	*261.6347*	*0.4070*	*0.8426*	*178.1001*
3	*0.3916*	*1.4331*	*299.9791*	*0.4589*	*1.0757*	*296.2747*
4	*0.4183*	*1.2393*	*357.7787*	*0.4637*	*0.9258*	*253.9949*
5	*0.3418*	*1.4411*	*321.3318*	*0.3574*	*1.3356*	*230.0798*
6	*0.3207*	*1.4276*	*300.1871*	*0.3452*	*1.1965*	*195.4725*
7	*0.3318*	*1.6370*	*279.5559*	*0.3160*	*1.4169*	*172.9545*
8	*0.3002*	*1.3821*	*254.5392*	*0.3347*	*1.4981*	*162.3985*
9	*0.3166*	*1.3638*	*257.7128*	*0.3221*	*1.3436*	*144.2637*
10	*0.2958*	*1.5458*	*256.1853*	*0.3159*	*1.3301*	*133.9074*

MethodsX
Open access
Article Template

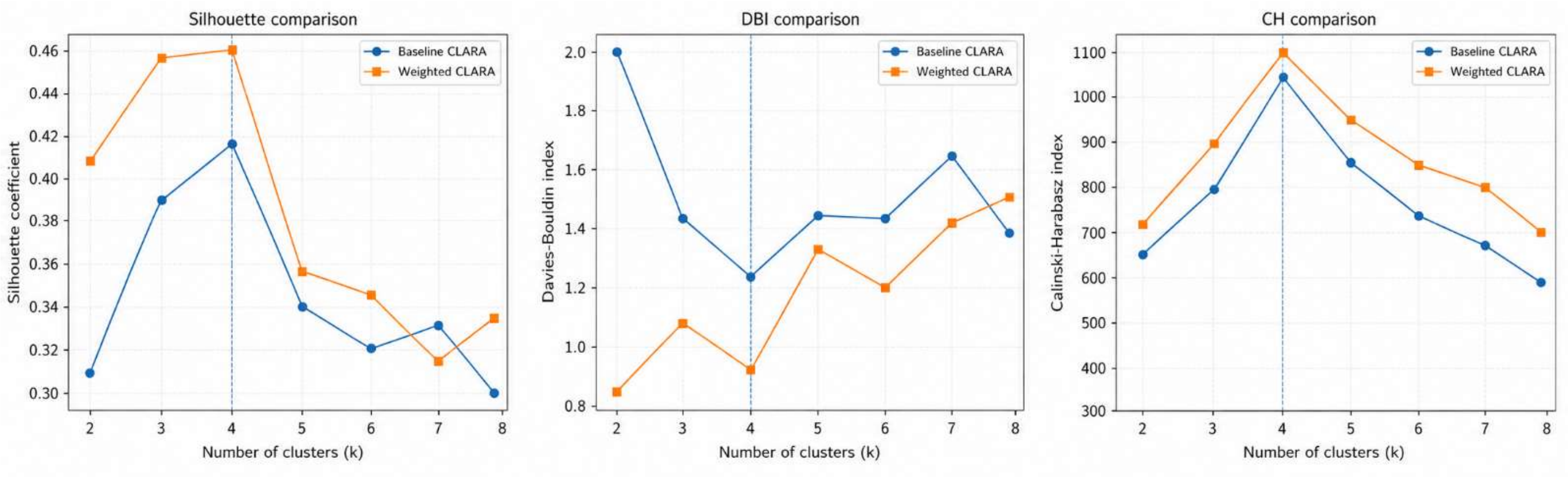

Figure 4. *Baseline and weighted CLARA validation across candidate cluster numbers.*

D. Top-N validation and selection

The Top-N procedure held k = 4 constant and successively removed lower-weight features. Under the same pre-specified selection rule, Top-4, comprising production capacity, assets, annual revenue, and workforce, was selected because it achieved the highest Silhouette Coefficient (0.5588) and the lowest medoid-based DBI (0.6181), while also producing a high Calinski-Harabasz Index value (992.3958). Compared with baseline CLARA at k = 4, SC increased by 33.59%, DBI decreased by 50.13%, and CH increased from 357.7787 to 992.3958. Although the Top-3 configuration produced a higher CH value, it had a marginally lower SC and a substantially higher DBI, indicating that dropping workforce removed useful separation information. Therefore, Top-4 was retained as the most balanced and interpretable configuration across cohesion, separation, compactness, and substantive feature relevance.

Table 5. *Top-N feature selection results at k = 4.*

Scenario	***Selected feature set***	***Silhouette coefficient***	***Medoid-based DBI***	***Calinski-Harabasz Index***
Top-8	*All eight features*	*0.4637*	*0.9258*	*253.9949*
Top-7	*All except Formal business registration availability*	*0.4653*	*0.9141*	*288.4188*
Top-6	*All except Formal business registration availability and social media*	*0.4802*	*0.8681*	*324.9926*
Top-5	Production capacity, assets, annual revenue, workforce, and marketplace use	*0.5070*	*0.9295*	*610.6572*
Top-4	*Production capacity, assets, revenue, workforce*	*0.5588*	*0.6181*	*992.3958*
Top-3	*Production capacity, assets, revenue*	*0.5555*	*0.8772*	*1214.3638*

E. Interpretability check: cluster profiles and ranking boundary

The selected Top-4 model generated four clusters of different sizes. Cluster profiles provide an interpretability check; they should be examined alongside, rather than replaced by, internal metrics. The results suggest a small-scale cluster, a workforce-and-capacity cluster, a financially strong cluster, and a high-output/lean-workforce cluster. These labels are descriptive and should be validated with local domain experts before use in policy or program design.

Table 6. *Average profile of the selected Top-4 clusters.*

MethodsX
Open access
Article Template

Cluster	n (%)	Workforce	Production capacity	Revenue/year	Assets
1	534 (50.9%)	6.98	1,203	109,621,395	99,599,485
2	145 (13.8%)	29.51	7,486	427,525,000	337,107,931
3	274 (26.1%)	7.02	1,041	791,286,004	765,337,591
4	96 (9.2%)	4.00	10,268	198,036,458	205,406,770

TOPSIS was then applied separately within each of the four selected clusters. Because the selected attributes are benefit criteria, the resulting score represents relative economic-operational readiness among comparable MSMEs within the same cluster, not the level of support need. Therefore, the cluster label should be interpreted first, followed by the within-cluster TOPSIS rank.

F. Within-cluster TOPSIS ranking demonstration

After the best clustering configuration was selected, TOPSIS was applied separately within each cluster using the four selected benefit criteria: production capacity, annual revenue, assets, and workforce. The ranking was conducted within each cluster to ensure that each MSME was compared only with comparable peer groups. Therefore, TOPSIS scores should not be compared across different clusters. Table 7 presents the highest- and lowest-ranked MSMEs in each cluster. The complete ranking output is supplied in the supplementary materials.

Table 7. *Summary of highest and lowest within-cluster TOPSIS scores for the selected Top-4 CLARA configuration.*

Cluster	Rank position	MSME code	TOPSIS score
1	Highest	C1-MSME-001	0.6629
1	Lowest	C1-MSME-534	0.0196
2	Highest	C2-MSME-001	0.9342
2	Lowest	C2-MSME-145	0.1287
3	Highest	C3-MSME-001	0.7285
3	Lowest	C3-MSME-274	0.0951
4	Highest	C4-MSME-001	0.7166
4	Lowest	C4-MSME-096	0.0523

Table 7 shows the highest and lowest TOPSIS scores within each selected cluster. The complete ranking output is supplied in the supplementary materials. TOPSIS scores are interpreted only within the same cluster because each cluster has its own normalization and ideal solutions.

G. Recommended robustness protocol for future applications

The demonstrated validation evaluates candidate cluster numbers k=2,…,10 and the specified Top-N feature configurations. For full deployment or application to a new dataset, the following robustness checks should be completed and reported:

1. *Repeat CLARA across at least 20–30 random seeds and report mean ± standard deviation of SC, DBI, and pairwise cluster agreement measures such as Adjusted Rand Index (ARI) or Variation of Information (VI) across runs.*
2. *Vary the CLARA sample fraction, for example 10%, 25%, and 40%, while keeping the minimum sample-size condition explicit.*
3. *Compare the selected model against at least one alternative mixed-data approach, such as PAM with weighted Gower distance, k-prototypes, or a model-based mixed-data method where assumptions are appropriate [32], [33], [34].*
4. *Inspect the influence of outlier handling by repeating analysis with no winsorization and with an alternative robust scaling policy.*

5. *Conduct a domain-expert review of cluster labels and verify that the TOPSIS criterion directions match the intended decision construct.*

Limitations

This study demonstrates the procedure using cross-sectional MSME records from one Indonesian regency; therefore, the resulting cluster profiles should not be generalized directly to other locations. Entropy weights are data-dependent and represent observed information dispersion rather than policy importance, causal influence, or universal feature relevance. Future work may investigate hybrid weighting schemes that combine objective entropy-based weights with expert-defined or policy-driven priorities. The complete weighted Gower matrix has quadratic memory requirements as the number of records grows; future implementations may therefore explore block-wise or distributed processing to improve scalability for larger datasets. Finally, within-cluster TOPSIS scores represent relative economic-operational readiness under the declared benefit criteria and should not be interpreted as causal measures of future MSME growth or as direct measures of support need. The cross-sectional design also does not capture changes in MSME characteristics or transitions between clusters over time; future studies may therefore use longitudinal data to examine cluster transitions and changes in economic-operational readiness across periods.

Ethics statements

This study analyzed administrative MSME records obtained through an authorized local data custodian. Direct business-identifying fields were excluded from the modelling workflow or replaced with anonymous codes before data sharing. The study did not involve clinical intervention, biological samples, experimental manipulation of human participants, or collection of sensitive personal data. The supplementary package contains an anonymized/de-identified version of the MSME dataset and does not contain direct business identifiers or the non-anonymized source records

CRediT author statement

***Yeni Kustiyahningsih:** Conceptualization, Methodology, Investigation, Data curation, Project administration, Writing – original draft. **Eza Rahmanita:** Data curation, Validation, Writing – review & editing. **Miswanto:** Validation, review. **Imamah:** Validation, Writing – review & editing. **Herdiyanti Fifin Purwaningrum:** Data curation, Visualization. **Firdausi Putri Cahyani:** Visualization, editing. **Deshinta Arrova Dewi:** Validation, review & editing. **Bain Khusnul Khotimah:** Methodology, Validation. **Jaka Purnama:** Data curation, Validation.*

Acknowledgments

This research was supported by the Institute for Research and Community Service (LPPM), University of Trunodjoyo Madura, Indonesia. The authors sincerely thank the relevant MSME data custodian, the Department of Cooperatives, Industry, and Trade (Diskoperindag) of Sampang Regency, and Madura MSME stakeholders for their support, contextual insights, and cooperation during the development and validation of the proposed decision-support procedure.

Declaration of interests

☒ The authors declare that they have no known competing financial interests or personal relationships that could have appeared to influence the work reported in this paper.

☐ The authors declare the following financial interests/personal relationships which may be considered as potential competing interests:

Supplementary material and/or additional information [OPTIONAL]

Supplementary materials supplied with this manuscript include entropy_clara_topsis.ipynb, dataset_umkm_sampang_25new.xlsx, hasil_all_clustering_topsis.xlsx, requirements.txt, README_TOPSIS.md, and TOPSIS_IMPLEMENTATION_GUIDE.md. These files contain the Jupyter Notebook implementation, anonymized/de-

identified empirical MSME dataset, dependency specifications, reproducibility instructions, TOPSIS implementation guide, and combined clustering–TOPSIS results. Direct business identifiers are not included; identifying fields were removed or replaced before submission.

References

[1] M. Jánošová, A. Lang, P. Budíková, E. Schubert, and V. Dohnal, "Advancing the PAM Algorithm to Semi-supervised k-Medoids Clustering," in Similarity Search and Applications (SISAP 2024), 2025, pp. 223–237, doi: 10.1007/978-3-031-75823-2_19.

[2] A. Grané and F. Scielzo-Ortiz, "New distance-based robust clustering algorithms for large mixed-type data," Adv. Data Anal. Classif., 2026, doi: 10.1007/s11634-026-00701-9.

[3] E. Roszkowska, M. Filipowicz-Chomko, A. Łyczkowska-Hańćkowiak, and E. Majewska, "Extended Hellwig's Method Utilizing Entropy-Based Weights and Mahalanobis Distance: Applications in Evaluating Sustainable Development in the Education Area," Entropy, vol. 26, no. 3, art. 197, 2024, doi: 10.3390/e26030197.

[4] Duanjiao Li, Yun Chen, Wenxing Sun, Yuhui Chen, Junwen Yao, Hua Ye, Jianguo Zhang. Subspace and metric space learning for coqualitative data clustering[J]. Electronic Research Archive, 2026, 34(6): 4191-4215. doi: 10.3934/era.2026188

[5] A. K. Kar, A. C. Mishra, and S. K. Mohanty, "EDMIX: an entropy-based dissimilarity measure to cluster mixed data comprising of numerical–nominal–ordinal attributes," Knowl. Inf. Syst., vol. 67, pp. 3023–3045, 2025, doi: 10.1007/s10115-024-02319-9.

[6] C.-L. Hwang and K. Yoon, Multiple Attribute Decision Making, vol. 186. Berlin, Heidelberg: Springer Berlin Heidelberg, 1981. doi: 10.1007/978-3-642-48318-9.

[7] A. I. Nor, "Small and medium-sized enterprises: A tool for socioeconomic development," Int. J. Adv. Appl. Sci., vol. 11, no. 12, pp. 116–128, Dec. 2024, doi: 10.21833/ijaas.2024.12.014.

[8] M. I. Firmansyah, Y. Kustiyahningsih, E. Rahmanita, M. S. Abidin, and B. D. Satoto, "Optimization of MSMEs Clustering in Sampang District Using K-Medoids Method and Silhouette Coefficient Method," Teknika, vol. 14, no. 1, pp. 1–8, Mar. 2025, doi: 10.34148/teknika.v14i1.1116.

[9] C. C. Astuti, B. W. Otok, and S. Andari, "Segmentation of partial least squares structural equation modelling using kernel K-means clustering (PLS SEM KKC)," MethodsX, vol. 15, Dec. 2025, doi: 10.1016/j.mex.2025.103570.

[10] A. Grané and F. Scielzo-Ortiz, "New distance-based robust clustering algorithms for large mixed-type data," Adv. Data Anal. Classif., 2026, doi: 10.1007/s11634-026-00701-9.

[11] Z. Huang, "Extensions to the k-Means Algorithm for Clustering Large Data Sets with Categorical Values," 1998. Data Mining and Knowledge Discovery, vol. 2, no. 3, pp. 283–304, 1998, doi: 10.1023/A:1009769707641.

[12] E. Costa, I. Papatsouma, and A. Markos, "Benchmarking Distance-Based Partitioning Methods for Mixed-Type Data," Adv. Data Anal. Classif., vol. 17, no. 3, pp. 701–724, 2023, doi: 10.1007/s11634-022-00521-7.

[13] P. Liu, H. Yuan, Y. Ning, B. Chakraborty, N. Liu, and M. A. Peres, "A modified and weighted Gower distance-based clustering analysis for mixed type data: a simulation and empirical analyses," BMC Med. Res. Methodol., vol. 24, no. 1, p. 305, Dec. 2024, doi: 10.1186/s12874-024-02427-8.

[14] D. Baviskar et al., "A sentiment-driven framework for early detection of emerging business trends through multi-platform social media analytics," MethodsX, vol. 16, Jun. 2026, doi: 10.1016/j.mex.2026.103874.

[15] M. Guo et al., "Normal Workflow and Key Strategies for Data Cleaning Toward Real-World Data: Viewpoint," Interact. J. Med. Res., vol. 12, p. e44310, Sep. 2023, doi: 10.2196/44310.

[16] R. Aschenbruck, G. Szepannek, and A. F. X. Wilhelm, "Imputation Strategies for Clustering Mixed-Type Data with Missing Values," J. Classif., vol. 40, pp. 2–24, 2023, doi: 10.1007/s00357-022-09422-y.

[17] A. Abuzaid and E. Alkronz, "A comparative study on univariate outlier winsorization methods in data science context," Ital. J. Appl. Stat., vol. 36, no. 1, pp. 85–99, 2024, doi: 10.26398/IJAS.0036-004.

[18] C. Wongoutong, "The impact of neglecting feature scaling in k-means clustering," PLoS One, vol. 19, no. 12, p. e0310839, Dec. 2024, doi: 10.1371/journal.pone.0310839.

[19] M. Ziemann, P. Poulain, and A. Bora, "The five pillars of computational reproducibility: bioinformatics and beyond," Brief. Bioinform., vol. 24, no. 6, art. bbad375, 2023, doi: 10.1093/bib/bbad375.

[20] E. Roszkowska, M. Filipowicz-Chomko, A. Łyczkowska-Hańćkowiak, and E. Majewska, "Extended Hellwig's Method Utilizing Entropy-Based Weights and Mahalanobis Distance: Applications in Evaluating Sustainable Development in the Education Area," Entropy, vol. 26, no. 3, art. 197, 2024, doi: 10.3390/e26030197.

[21] A. Grané and F. Scielzo-Ortiz, "On generalized Gower distance for mixed-type data: extensive simulation study and new software tools," SORT, vol. 49, no. 2, pp. 65–96, 2025, doi: 10.57645/20.8080.02.15.

[22] A. Moslemi, "A tutorial-based survey on feature selection: Recent advancements on feature selection," Eng. Appl. Artif. Intell., vol. 126, part D, art. 107136, Oct. 2023, doi: 10.1016/j.engappai.2023.107136.

[23] Z. Xu, F. Yang, H. Wang, J. Sun, H. Zhu, S. Wang, and Y. Zhang, "CGUFS: A clustering-guided unsupervised feature selection algorithm for gene expression data," J. King Saud Univ. Comput. Inf. Sci., vol. 35, no. 9, art. 101731, Oct. 2023, doi: 10.1016/j.jksuci.2023.101731.

[24] X. Du, "A Robust and High-Dimensional Clustering Algorithm Based on Feature Weight and Entropy," Entropy, vol. 25, no. 3, art. 510, 2023, doi: 10.3390/e25030510.

[25] R. Cafaro, B. Cardone, and F. Di Martino, "A Novel Two-Level Entropy-Weighted Fuzzy C-Means Algorithm and Its Application for Classifying Urban Patterns by Residential Building Characteristics," Symmetry, vol. 18, no. 5, art. 807, 2026, doi: 10.3390/sym18050807.

[26] L. Lenssen and E. Schubert, "Medoid Silhouette clustering with automatic cluster number selection," Inf. Syst., vol. 120, art. 102290, Feb. 2024, doi: 10.1016/j.is.2023.102290.

[27] A. Anfossi and D. Chicco, "An easy guide to the Davies-Bouldin index for unsupervised internal clustering evaluation," Discov. Comput., 2026, doi: 10.1007/s10791-026-10094-0.

[28] M. Baragilly and H. Gabr, "High-Dimensional BWDM: A Robust Nonparametric Clustering Validation Index for Large-Scale Data," arXiv:2510.14145 [stat.ME], 2025.

[29] W. McKinney, "Data Structures for Statistical Computing in Python," 2010, pp. 56–61. doi: 10.25080/Majora-92bf1922-00a.

[30] C. R. Harris et al., "Array programming with NumPy," Nature, vol. 585, no. 7825, pp. 357–362, Sep. 2020, doi: 10.1038/s41586-020-2649-2.

[31] F. Pedregosa et al., "Scikit-learn: Machine Learning in Python," 2011. [Online]. Available: http://scikit-learn.sourceforge.net.

[32] G. Szepannek, R. Aschenbruck, and A. Wilhelm, "Clustering large mixed-type data with ordinal variables," Adv. Data Anal. Classif., vol. 19, no. 3, pp. 749–767, Sep. 2025, doi: 10.1007/s11634-024-00595-5.

[33] E. Costa, I. Papatsouma, and A. Markos, "A Deterministic Information Bottleneck Method for Clustering Mixed-Type Data," in Data Science, Classification, and Artificial Intelligence for Modeling Decision Making, Springer, Cham, 2025, doi: 10.1007/978-3-031-85870-3_9.

[34] R. Aschenbruck, G. Szepannek, and A. F. X. Wilhelm, "Initialization strategies for clustering mixed-type data with the k-prototypes algorithm," Adv. Data Anal. Classif., 2025, doi: 10.1007/s11634-025-00639-4.
